# Video-mediastinoscopy assisted fish bone extraction and superior Medistinal abscess debridement

**DOI:** 10.1186/s13019-018-0732-7

**Published:** 2018-05-15

**Authors:** Jun Wang, Wei Bing Wu, Liang Chen, Quan Zhu

**Affiliations:** Department of Thoracic Surgery, The First Affiliated Hospital of Nanjing Medical University, Nanjing, China

**Keywords:** Esophageal foreign body, Mediastinum abscess, Video-Mediastinoscopy

## Abstract

**Background:**

Mediastinum abscess caused by sharp esophageal foreign body perforation usually needs surgical treatment, and the surgical procedures vary according to size of perforation and scope of abscess, etc. For special case with small esophageal mucosal crevasse and focal abscess confined to mediastinum, minimally invasive surgery with guidance of video-mediastinoscopy would be an alternative method, however, application of video-mediastinoscopy in this life-threatening situation was rarely reported.

**Case presentation:**

One patient with detention of fish bone stuck in the esophagus developed systemic inflammatory response syndrome. Computed tomography results revealed that two high-density foreign bodies migrated extraluminally and caused abscess confined in the mediastinum. Multidisplinary collaborative efforts of anesthesiology, gastroenterology and thoracic surgery were made to optimize the therapeutic process. By taking advantages of wide working channel and better exposure of video-mediastinoscopy, two sharp fish bones were removed with minimal risk of injuring adjacent important tissues, furthermore, complete debridement of the abscess and precise drainage tube indwelling was achieved simultaneously. Postoperative comprehensive therapy including anti-infection and nutrition support guaranteed a smooth transition of perioperative period.

**Conclusion:**

This is the first report on application of video-mediastinoscopy in removing two fish bones that migrated extraluminally and debridement of the abscess caused by esophagus perforation with minimal injury risk, which offer a safe and effective minimal invasive method for specific cases.

## Background

Detention of sharp esophageal foreign body often causes esophagus perforation and mediastinal abscess [1]. Under severe inflammation situation, the complexity of the cervical anatomy and unsatisfactory surgical exposure pose certain difficulty for surgeons to extract the foreign body and clear the abscess completely. For such a dilemma,multidisplinary collaborative efforts of anesthesiology, gastroenterology and thoracic surgery are imperative. Also, a suitable surgical strategy, with the appliance of endoscopic technique in intervention of foreign body and abscess confined in the mediastinum, will guarantee the safety of operation and lead to a more satisfactory result.

## Case presentation

**Fig. 1:**
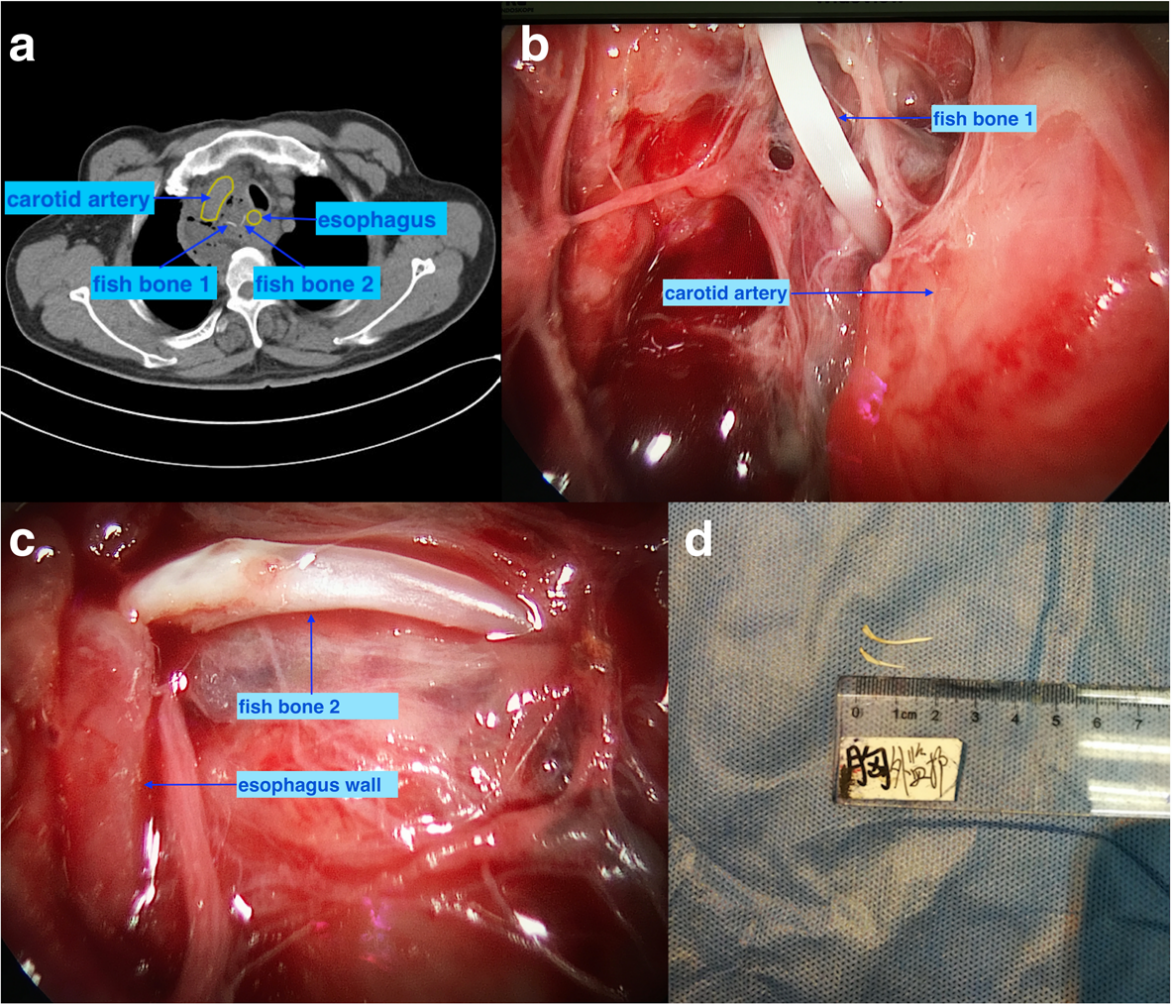
Preoperative, intraoperative and postoperative findings of fish bones. Preoperative, intraoperative and postoperative findings of fish bones. **a** CT result showed that an abscess confined to the right mediastinal pleural with two high density foreign bodies in the abscess at the level of the sternum angle. **b** Under video-mediastinoscopy, fish bone 1 was located at the medial margin of the carotid artery. **c** Under video-mediastinoscopy, fish bone 2 was located on the outside of the esophagus wall. **d** Two fish bones after being taken out

A 63-year-old male patient was transferred to the emergency department of the first affiliated hospital of Nanjing Medical University for esophageal foreign body. Two weeks ago, he swallowed fish bone and it got stuck, after attempt of pushing downward by bolus, the foreign body sensation aggravated gradually in the next few days, and the local community hospital initiated antibiotics therapy on the 3rd day without laryngoscope and gastroscopy examination for local poor medical condition. Until referral on the 12th day, symptoms showed no signs of decreasing. On admission, the patient couldn’t lie down due to a swollen neck with activity limitation, and body temperature was 39 °C; blood pressure, 139/82 mmHg; hemoglobin 11.5 g and white blood cell count 20,200. Contrast-enhanced CT results showed an abscess confined to right mediastinal pleural with two high density foreign bodies in the abscess at the level of the sternum angle, one located the medial margin of the carotid artery and the other at outside of the esophagus wall (Fig. 1 a).

Surgical scheme was made to adopt a single lumen intubation, a horizontal position, with upper back elevated to suitable height avoiding neck overextension and possible injury of fish bones to the adjacent carotid artery. After anesthesia, the location and diameter of perforation was explored by a gastroenterologist with endoscopy, which showed a rim of compressed fibrous tissue protruding from the esophageal mucosa 24 cm from the incisor. Nasogastric decompression tube and nasointestinal feeding tube were placed, while stent was needless since no obvious crevice was found. Subsequently, the thoracic surgeons probed the abscess with an incision anterior to the border of sterno-mastoid muscle. After exposing the deep layer of cervical fascia, the abscess wall with high tension was palpable. After abscess cavity decompression and pus drawing for a bacteriological examination with injector puncture, the abscess wall was cut and the pus was cleared with further suction. In consideration of the sharp fish bone adjacent to the carotid artery and the deep abscess extending to the mediastinum, which caused huge risk of bleeding and difficulty of exposure, video-mediastinoscopy was applied via the abscess wall incision. Two fish bones with locations coincident to preoperative CT results were displayed clearly on the screen (Fig. 1 b, c). With a grasper through the wide operating channel of the video-mediastinoscopy, fish bones were removed carefully, then,the abscess septations were debrided completely and flushed with diluted iodine, hydrogen peroxide and saline in sequence. Three drainage tubes were embedded at the top, center and bottom of the abscess.

Postoperative comprehensive therapy included anti-infection and nutrition support. Pus culture was positive for pseudomonas aeruginosa, which is sensitive to sulperazone. The patient received intravenous drip for 7 days until the body temperature and blood routine returned to normal. Continuous irrigation with diluted iodine lasted for 7 days until liquid drainage disappeared. On postoperative day 14, after upper gastroenterography and CT confirmed satisfactory closure of the abscess cavity without contrast agent leakage and all routine examinations recovered to normal, all indwelling tubes were removed. Enteral feeding was then initiated and the patient was discharged without any problems on postoperative day 16. Follow-up results on postoperative day 30 showed no problem.

## Discussion and conclusions

Results of esophageal foreign body varied according to the foreign body shape, size, location and compression time. Multidisciplinary efforts of radiologist, gastroenterologist, anesthesiologist and thoracic surgeon were preferred to make preoperative planning for best the solution. In this case, delayed treatment led to perforation and subsequent focal abscess confined within the deep cervical fascia without breaking into the chest cavity. After the gastroenterologist confirmed that the esophagus perforation had been covered by fibrous tissue, by taking advantages of wide working channel and better exposure of video-mediastinoscopy, sharp foreign body was removed with minimal risk of injuring adjacent important tissues, furthermore, complete debridement of the abscess and precise drainage tube indwelling was achieved simultaneously. Albeit with above advantages, this procedure is not without risks. When placing the mediastinoscopy instrument and operating in an inflammatory environment, one has to be ever vigilant. Major complications including injury to blood vessels, tracheobronchial tree, recurrent laryngeal nerve and esophagus were reported sporadically during past years [2]. Precise operation and intimate postoperative observation is warranted to eliminate those iatrogenic injuries. In this case, careful intraoperative exploration avoided residual of the fish bones. When the foreign body is fixed in tissues, brute force pulling is prohibitive, and the fish bone can be cut into half with scissor and then pulled out. Possible injuries to adjacent tissues should be explored carefully and disposed properly. So far, there are only three reports of foreign body removal under mediastinoscope, two cases of bullet in the superior mediastinum and one case of teeth prosthesis in upper esophagus narrowing, all without esophagus perforation [3–5]. This is the first report on application of video-mediastinoscopy in removing two fish bones that migrated extraluminally and debridement of the abscess caused by esophagus perforation with minimal injury risk, which will provide a safe and effective minimal invasive method for specific esophageal foreign body cases. Also, multidisciplinary collaboration of optimizing the process to achieve an ideal result is worth spreading.
